# The effect of grazing on winter survival of midday gerbil (*Meriones meridianus*) of different genders

**DOI:** 10.1002/ece3.6870

**Published:** 2020-10-15

**Authors:** Su‐Wen Yang, Shuai Yuan, Xiao‐Dong Wu, Rong Zhang, Xiu‐Xian Yue, Yu Ji, Lin‐Lin Li, Xin Li, He‐Ping Fu

**Affiliations:** ^1^ College of Grassland, Resources and Environment Inner Mongolia Agricultural University Hohhot China; ^2^ Key Laboratory of Grassland Resources of the Ministry of Education Key Laboratory of Forage Cultivation, Processing and High Efficient Utilization of the Ministry of Agriculture and Rural Affairs Hohhot China; ^3^ Rodent Research Center Inner Mongolia Agricultural University Hohhot China; ^4^ Institute of Forestry Monitoring and Planning of Inner Mongolia Autonomous Region Hohhot China

**Keywords:** food availability, gender difference, grazing, overwinter survival

## Abstract

The objective of this study was to investigate the effects of grazing on midday gerbil (*Meriones meridianus*) population characteristics and survival of animals of different genders. The experiment used a randomized complete block design and was conducted in Alxa Left Banner, Inner Mongolia, China, in 2002 (The agricultural reclamation plots set up in 1994). From April 2006 to October 2010, midday gerbils were live‐trapped in 3 light grazing plots, 3 overgrazed plots, and 3 grazing exclusion plots. The quantity of vegetation was investigated in the two different grazing intensity areas and grazing exclusion area to determine the relationship between gerbils and plant food availability. The results suggested that there was higher gerbil density, individual body mass, and daily body mass growth rate in the grazing exclusion sites than the other sites across the whole year. Females had higher survival in grazing exclusion areas than in other treatments, but the males’ survival showed the opposite pattern. Our results indicated that grazing negatively influenced the midday gerbil population by reducing food availability. Grazing influenced the survival rates of male midday gerbils positively, but had negative effects on females. The reason for gendered differences in survival rates of midday gerbils requires further investigation.

## Graphical abstract

The objective of this study was to investigate the effects of grazing on Midday gerbil (*Meriones meridianus*) population characteristics and overwinter survival of animals of different genders.

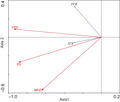

## 1 INTRODUCTION

Livestock grazing is the most common land use worldwide (Kemp et al., 2013). Grazing induced changes in plant communities and soil properties affect the dynamics of small‐mammal populations and communities (Fuhlendorf et al., 2010; Keesing, 1998a; Li et al., 2016; Saetnan et al., 2012; Steen et al., 2005; Wang et al., 2019). For example, wild and domestic ungulate grazing may affect small mammals through changing grassland ecosystems and food availability (Keesing, 1998b; Milchunas et al., 1998). Food availability, for example, plays a key role in the fitness, home range size, predation risk, physiology, behavior, life history, and overwinter survival of small mammals (Brown et al., 2004; Liu et al., 2011; Schoepf et al., 2015). The food availability hypothesis posits that grazing changes food plant availability and food plant quality for small herbivorous mammals and thus may affect their population size, survival, body mass, and reproduction (Jon et al., 1993; Keesing, 1998b; Li et al., 2016; Schmidt et al., 2005). However, in arid ecosystems, grazing may produce also positive effects on rodents depending on their ecology, and in a relatively dry environment, grazing will drive the rodent community to develop into a community with fewer dominant species (Jones et al., 2003; Tabeni & Ojeda, 2005). So the grazing affect the life of rodent may influenced by many external condition and different object. Most studies have assessed the responses of rodent density or population growth rates to grazing (Cockburn & Lidicker, 1983; Klemolaet al., 2000; Rosi et al., 2009), while few studies have investigated the effects of grazing on demographic rates such as survival probabilities of small mammals with different genders (Keesing, 1998b; Korslund & Steen, 2006; Yuan et al., 2018).

Because food is scarce, the environment is harsh, and winter is a difficult period for mammalian nonhibernators in high northern latitudes (Coltrane & Barboza, 2010; Solonen, 2006). Mammals cope with this predictable period of food scarcity by caching food in the late fall and/or hibernating (Geiser & Ruf, 1995; Turbill et al., 2011). Food caching is a widespread behavioral adaptation used primarily by nonmigratory and nonhibernating species to store food for future use during periods of low resource availability or uncertain environmental dynamics (Sutton et al., 2016). A caching specie relies on stored food for survival during periods of limited food availability and, in some cases, for reproduction, and food quality could have major downstream effects on fitness and population dynamics (Sutton et al., 2016). Caching species can generally be divided into two classes based on the cache duration: “short‐term hoarders” and “long‐term hoarders” (Vander Wall, 1990). The midday gerbil (*Meriones meridianus*) is an example of the latter class. This caching species engages in intense periods of caching, usually in the late summer or fall (Degange et al., 1989; Jansson et al., 1981; Vander Wall, 1990). Both autumn and winter food availability may play crucial roles in the overwinter survival of midday gerbils.

Midday gerbil spans the major arid and semiarid biogeographic regions in northern of China and northwest China as far west as the Caspian Sea and occur in open desert habitats on stabilized, semistabilized, or nonstabilized sands (Luo et al., 2000; Rogovin, 2007; Shenbrot et al., 1999). The range of the midday gerbil covers a large region of the Eurasian arid zone. This superspecies is characterized by remarkably high geographical variation (Gromov & Erbajeva, 1995; Heptner, 1968). The type specimen was produced in the lower reaches of the Urals in Kazakhstan (Pallas, 1773). Midday gerbil is a small‐sized desert dewelling rodent. The average weight of males was 52.26 ± 1.00 g and females was 50.51 ± 0.98 g (Zhang et al., 2017). The duration of pregnancy in uncontrolled laboratory conditions was found to last 24–27 days, and new‐born gerbils weigh about 2.45 g (Özkurt et al., 2001). It has a mixed diet of seeds, insects, and green plant parts, but seeds dominate in the diet (Shenbrot et al., 1999; Rogovin, 2007). The reproduction period of midday gerbil, which occurs two or three times per year, is from late March to early November in our study area, and breeding activity reaches its maximum in July (Jin et al., 2009; Yang et al., 2017; Zhou et al., 1999). In general, a lower population density occurs in late autumn, and is higher in spring (Zhou et al., 2012). Thus, the breeding period may start in a season without new food resources, and both the food stored and vegetation situation may play a key role in its first breeding activity every year. Midday gerbils mainly feed on seeds, leaves, stems and insects, and food composition may vary depending on conditions in different microhabitats. Midday gerbils engage in food caching behavior in late autumn, but since this is insufficient to provide food for the whole winter, they go out of their borrows in winter (Song & Liu, 1984). Reproductive activity of midday gerbils starts in early spring when the environment could not provide any new foods, so the amount of stored food in winter will affect reproduction. In some species, females will reduce reproductive costs in harsh external conditions to ensure survival over the winter (Doonan & Slade, 1995). This specie is typically nocturnally active, and we never caught any specimens during the day when we live traps were set at day and night.

The purpose of our study was to test how livestock grazing affects the density and body mass of midday gerbils in early spring and late autumn, and the overwinter survival of gerbils. In particular, we were interested in whether there are gendered differences in these effects. We proposed three hypotheses. First, compared to overgrazed and lightly grazed areas, grazing exclusion should induce higher male and female gerbil population density both in early spring and late autumn. Second, compared to overgrazed and lightly grazed areas, grazing exclusion should induce higher body mass and body mass daily growth rate in both male and female gerbils. These effects could occur because grazing can affect plant productivity and ecosystem functions (Wang et al., 2019). Because females show a positive correlation between survival probability and body mass while males do not (Korslund & Steen, 2006), our third hypothesis was that, compared to overgrazing and lightly grazed areas, grazing exclusion would induce higher winter survival rates of gerbils.

## 2 MATERIALS AND METHODS

### 2.1 Study area

This study was conducted in southern Alxa Left Banner at the eastern edge of the Tengger Desert, Inner Mongolia, China, (E104°10ʹ–105°30ʹ, N37°24ʹ–38°25ʹ) from April 2006 to October 2010. Our study area has a continental climate with cold and dry winters and warm summers. Temperature range from −36 to 42°C with an annual mean of 8.3°C. Annual precipitation ranges from 45 to 215 mm, but about 70% falls from June to September. Potential evaporation ranges from 3000 to 4700 mm, and the annual frost‐free period is 156 days. Approximately 5%–15% of the ground is covered with shrubs, forbs, and some gramineous plants. Shrubs mainly consist of *Zygophyllum xanthoxylon*, *Nitraria tangutorum*, *Caragana brachypoda*, *Ceratoides latens*, *Oxytropis aciphylla*, *Artemisia sphaerocephala,* and *Artemisia xerophytica*, with *Reaumuria soongorica* as the dominant species. The major grasses/forbs species are *Cleistogenes squarosa*, *Peqanum nigellastrum*, *Cynanchum komarovii*, *Salsola pestifer Suaeda glauca*, *Bassia dasyphylla*, *Corispermum mongolicum*, *Artemisia dubia, and Plantago lessingii* (Yuan et al., 2018). Midday gerbil, northern three‐toed jerboa (*Dipus sagitta*), and Mongolian five‐toed jerboa (*Allactaga sibirica*) are dominant small‐mammal species, and predators such as long‐eared hedgehog (*Hemiechinus auritus*), Eurasian eagle owl (*Bubo bubo*), Marbled polecat (*Vormela peregusna*), and Corsac fox (*Vulpes corsac*) are also present in the system (Wu & Fu, 2005; Xu, 2013).

This sample areas had been established at the beginning of this century. The experiment adopted a randomized block design with 3 blocks and 4 treatments (i.e., light grazing, overgrazing, grazed exclusion, and land reclamation) to study the dynamics of desert small mammals community under different grazing intensity. Each block is 240 ha, and each treatment unit is 60 ha. The agricultural reclamation plots previously had plant species similar to the ungrazed plots but were reclaimed in 1994 by planting saxaul (*Haloxylon ammodendron*), sunflowers, and maize. Before the study areas were set up (prior to 2002), every treatment blocks experienced the same grazing events. The treatments assessed in this study were overgrazing, light grazing, and grazing exclusion only, which were established with standard sheep fencing (110 cm high). In the overgrazing sites, sheep grazing intensity was close to prevailing grazing intensity in the local areas and was controlled within the range of 3.75 to 4.23 sheep per ha. In the light grazing sites, sheep grazing intensity was in line with Inner Mongolia government standards and was controlled within the range of 0.83 to 1.00 sheep per ha. A 7 × 8 gerbil trapping grid (0.96 ha) at a 15‐m intertrap distance was established at the center of each plot (60 ha). One wire‐mesh live trap (42 cm × 17 cm × 13 cm) was placed at each trap station (Yuan et al., 2018). A wooden protective box (15 cm × 7 cm × 10 cm) is placed inside each live cage to protect rodents from natural enemies, precipitation, or low temperatures.

### 2.2 Trapping of gerbils

We live‐traped midday gerbils from April to October, October 2006 to October 2010, on 4 consecutive days at 4‐week intervals. We did not trap during winter (from November to March) due to the low temperature. Traps were baited with fresh pignuts and checked twice (morning and afternoon) each day. Each captured individual was sexed and marked with a passive integrated transponder (PIT) tag with a unique identification number (ID) injected under the pelage. The sex, capture station, body mass, and reproductive condition of each captured individual were recorded. Males were considered in reproductive condition if they had scrotal testes. Females were considered reproductive if they possessed enlarged nipples surrounded with white mammary tissue, or a bulging abdomen. We classified gerbils as juveniles if they weighed <26 g and adults (subadult) if they weighed ≥26 g (Zhang et al., 2017), and we only captured 5 juvenile midday gerbils during the entire experiment. Therefore, in the data analysis of this study, we ignore the difference between larvae and adults. In order to minimize the impact of cage capture on midday gerbils, a wooden thermal insulation box was placed in the live cage to avoid the danger of midday gerbils from natural enemies, low temperature, and precipitation. The time to check the cage in the morning is generally before 6 o'clock in the morning. Other experiments (infrared camera monitoring) in the same study sites found that the gerbils continue to be active after 6 o'clock, so it will reduce the impact of the experiment on the gerbils. Snap trapping is a traditional survey method for long‐term studies of rodent populations and does not affect population dynamics (Christensen & Hörnfeldt, 2003; Hörnfeldt, 2004).

In all experiments, we carried out animal care and treatment in accordance with the guidelines issued by the Ethical Committee of Inner Mongolia Agricultural University. The committee requires that all researchers and students related to wildlife and experimental animals are certified in accordance with the requirements of the Institutional Animal Care and Use Committee of the Institute of Zoology, Chinese Academy of Sciences (IOZ11012).

### 2.3 Vegetation sampling

Vegetation sampling was carried out monthly from April to October during 2006 to 2010. We randomly placed 3 100‐m^2^ square sampling plots in each treatment unit to sample shrubs and randomly placed 3 1‐m^2^ quadrats in each 100‐m^2^ square plot to sample grasses and forbs. We estimated aboveground standing biomass of shrubs, grasses, and forbs by species (Yuan et al., 2018). An additional experiment on feeding behavioral observations of midday gerbils was conducted in 2017 to determine the food resource from all plants (Table S1). Preferred foods and potential food resources were divided according to the preference index (PI) (Batzli & Pitelka, 1983). Plant species from food resources were chosen to calculate total food biomass (TFB) and preferred food biomass (PFB) (Table S1).

### 2.4 Statistical analyses

The Cormack–Jolly–Seber (CJS) models were used as implemented in the program MARK 6.0 to estimate monthly apparent survival probabilities of the midday gerbils (White & Burnham, 1999). Overdispersion of our general model [ϕ(Grazing × Time) p(Grazing × Time)] was tested using the bootstrap goodness‐of‐fit method within MARK with 500 iterations. Parameters *φ* and *p* represent estimation of local survival probabilities and recapture probabilities, respectively. The test showed evidence of overdispersion with the variance inflation factor c‐hat of 1.20 (*p* = .13), 1.37 (*p* = .06), and 1.95 (*p* = .12) (Table S2). Thus, we used quasi‐Akaike's information criterion corrected for small sample size (QAICc) to select the most parsimonious and competing models of midday gerbil monthly survival (Burnham & Anderson, 2002). The most parsimonious model is the model with the lowest QAICc among all candidate models considered. A model with ∆QAICc < 2 was considered as a competing model (Burnham & Anderson, 2002). In a preliminary MARK analysis of the capture–recapture data by treatments, we found that the candidate models had gender effects (Table S3). Therefore, we assumed that the local survival probabilities of individuals would differ between the sexes. We built 15 candidate models including all possible models for the effects of treatment, time, and their interaction on survival probabilities and all possible models of the effects of time, treatment, and time–treatment interaction on recapture probabilities. If a model including treatment effects on survival probabilities was the most parsimonious model or a competing model, we concluded that sheep grazing significantly affected survival of the midday gerbil. A ninety‐five percent confidence interval (CI) was provided for survival probability. Nonoverlapping CIs were considered to be significantly different (Bieber et al., 2011).

We indexed the late autumn (October) and early spring (April) population density of midday gerbils from 2006 to 2010 using the minimum number known alive (MNKA) (Krebs, 1966; Hilborn, Redfield, & Krebs, 1976). All of the male and female adults in both grazing and nongrazing treatments were compared by body mass in late autumn. Daily proportional body mass growth rate (DPBMGR, i.e., growth rate) of a midday gerbil in late autumn was calculated as the difference in body mass between two successive trapping occasions divided by the initial body mass at the first trapping occasion and the number of days between the two trapping occasions (Agrell et al., 1992). All the data involved in this paper have been Shapiro–Wilk tested, in which body weight (*p >* .05, *n* = 30), growth rate (*p* > .05, *n = *28), and population density (*p* > .05, *n* = 30) all conform to the normal distribution. In addition, the sex ratio, total food biomass (TFB), and preferred food biomass (PFB) have been normalized (log(*n* + 1)) prior to the analysis.

A mixed‐model analysis of variance was performed on the data, with blocks and years set as random effects. Differences in the population density of gerbils, body mass, DPBMGR, and food resources between treatments in late autumn (October) or early spring (April) were tested using mixed‐effect models (PROC MIXD, SPSS 22.0) with a significance level of α = 0.05, using Tukey's test and the LSD test for means comparisons. The impact of environmental (TFB and PFB) variables on the population density of gerbils was tested with redundancy analysis (RDA) using the Monte Carlo Permutation Test processed by CANOCO 4.5 for Windows. Unless otherwise noted, data are presented as means ± one standard error of means (*SEM*). All the graphics were performed with SIGMAPLOT 12.0 (Systat Software, Inc., San Jose, USA).

## 3 RESULTS

### 3.1 Population density

**FIGURE 1:**
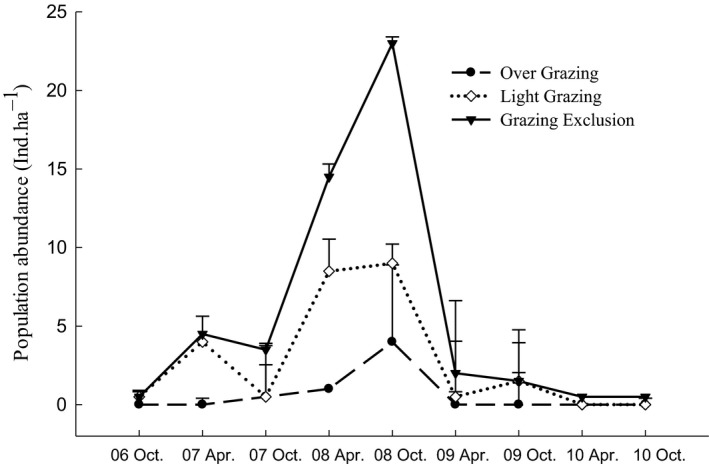
The population density of midday gerbils in overgrazing, light grazing, and grazing exclusion sites in April and October from 2006 to 2010 in Alxa Left Banner, Inner Mongolia, China

**FIGURE 2:**
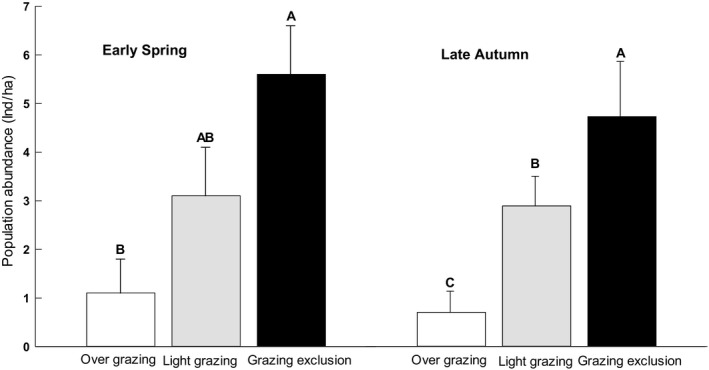
Mean population density ($\bar{x}$ ± *SE*) of midday gerbils captured from overgrazing, light grazing, and grazing exclusion sites in April and the previous October, from 2006 to 2010 in Alxa Left Banner, Inner Mongolia, China. Different letters (A, B, and C) indicate significant differences (*p* < .05)) among overgrazing, light grazing, and grazing exclusion sites, according to Tukey's test

A total of midday gerbil individuals, including 248 males (3 juveniles) and 217 females (2 juveniles), were captured, with 650 valid capture events in 47,040 trap days. The population density of midday gerbils dramatically varied with years, and a lower population density occurs in late autumn and a higher in spring (Figure 1). Grazing had a significant effect on midday gerbils population abundance in both early spring (*F*_2,7_ = 3.09, *p* = .019) and late autumn (*F*_2,7_ = 8.61, *p* = .017). Similarly, the year had a significant effect on the population of midday gerbils both in early spring (*F*_4,22_ = 2.98, *p* = .038) and late autumn (*F*_4,22_ = 8.281, *p* = .00, Table 1). The main reason for this result is that the population of midday gerbils is significant higher in 2008 than in other years. However, we did not detect a sex difference in gerbil abundance both in late autumn and early spring (*p *> .05, Table 1). Grazing exclusion significantly enhanced the gerbil population compared to the light and overgrazing treatments in both late autumn (*F*_2,27_ = 5.77, *p* = .011) and early spring (*F*_2,27_ = 5.40, *p* = .008; Figure 2). The light grazing treatment was not significantly different from the other two areas in late autumn.

**TABLE 1:** Source of variance (*F* and *P* values) from two‐way analysis of variance for the effects of sex and grazing on midday gerbil population density (*n* = 36) in late autumn (October) and early spring (April), on adult midday gerbils body mass (*n* = 195) in late autumn (October) and on adult midday gerbils daily proportional body mass growth rate (whole study period) (*n* = 27) from 2006 to 2010 in Alxa Left Banner, Inner Mongolia, China

Factor type	Factors	*df* (numerator, denominator)		*F*	*p*
Population density (Late Autumn)					
Stable factors	Grazing		2, 7	3.09	.019
	Sex		1, 7.4	0.10	.763
	Sex × Grazing		2, 7.6	0.02	.973
Random factor	Year		4, 22	8.281	.00
Population density (Early Spring)					
Stable factors	Grazing		2, 7	8.61	.017
	Sex		1, 7.48	0.21	.660
	Sex × Grazing		2, 7.29	0.14	.876
Random factor	Year		4, 22	2.98	.038
Body mass (Late Autumn)					
Stable factors	Grazing		2, 72	14.00	<.001
	Sex		1, 72	0.29	.59
	Grazing × Sex		2, 72	0.25	.774
Random factor	Year		4.22	10.76	.616
Daily proportional body mass growth rate					
Stable factors	Grazing		2, 21	1.602	.011
	Sex		1, 21	1.613	.21
	Grazing × Sex		2, 21	1.937	.155
Random factor	Year		–	–	–

### 3.2 Body mass and daily proportional body mass growth rate

**FIGURE 3:**
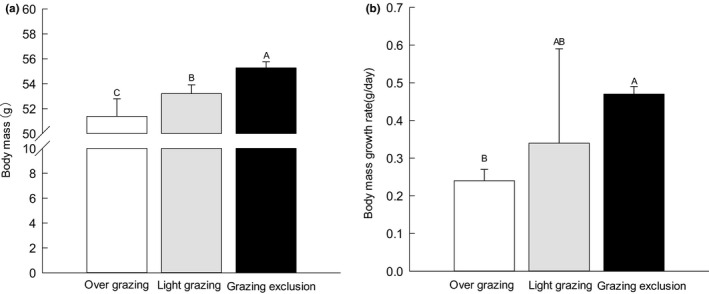
Mean adult body mass (a; $\bar{x}$ ± *SE*,) and daily proportional body mass growth rate (b; $\bar{x}$ ± *SE*) of midday gerbils captured from overgrazing, light grazing and grazing exclusion sites from late autumn 2006 to early spring 2010 in Alxa Left Banner, Inner Mongolia, China. Different letters (A, B, and C) indicate significant differences between overgrazing, light grazing, and grazing exclusion sites (*p* < .05), according to Tukey's test

Grazing treatments influenced late autumn body mass and daily proportional body mass growth rate of adult midday gerbils (*F*_2,72_ = 14.00, *p* = .001; *F*_2,21_ = 1.602, *p* = .011; Table 1). Midday gerbils in grazing exclusion had a significantly higher body mass than in light grazing sites and overgrazing sites (*F*_2,72_ = 52.80, *p* = .023; Figure 3a). Midday gerbils in the grazing exclusion sites had higher body mass growth rates than in overgrazing sites before overwintering (*F*_2,21_ = 3.39, *p* = .026; Figure 3b). There was no difference in body mass or body mass growth rate (*p* > .05, Table 1) between the two genders. There is no interyear difference between individual body mass (*p* > .05, Table 1). When analyzing the daily proportional body mass growth rate, most years have data missing (Especially in the overgrazing plot), so the impact of the year on body weight growth is not analyzed.

### 3.3 Survival probability

**FIGURE 4:**
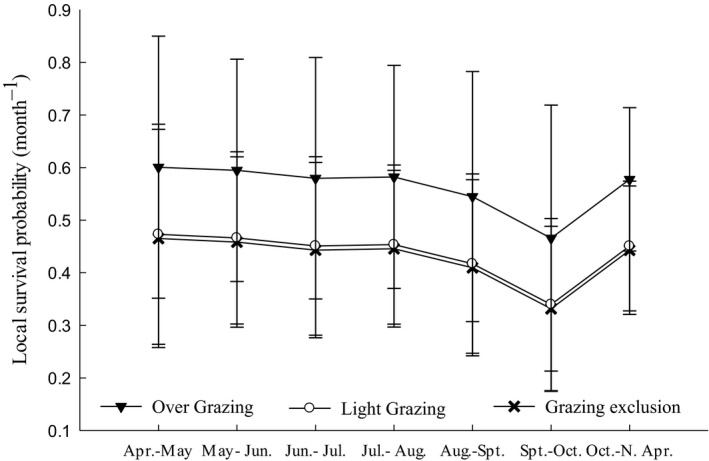
Monthly local survival probability for male midday gerbils ($\bar{x}$ ± *SE*, *n* = 270). Survival estimates are shown with 95% lower and upper confidence intervals (CI). The *x*‐axis indicates sample occasions. N Apr. represents April in the following year. Gerbils had lower survival rates in late autumn and did not significantly differ among years

**FIGURE 5:**
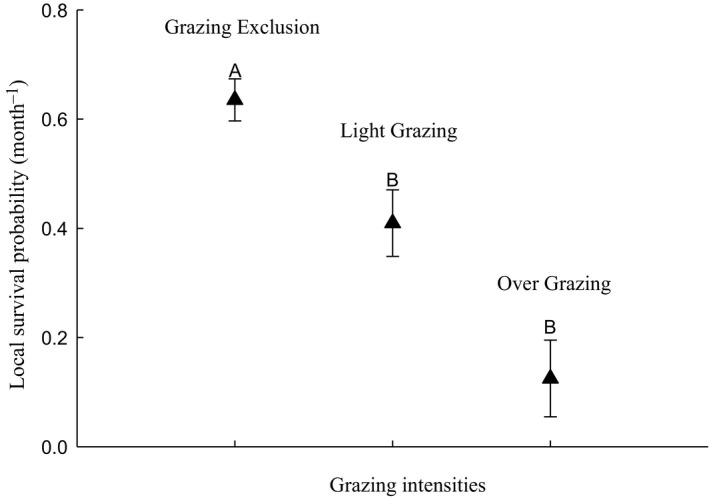
Monthly survival probabilities of female midday gerbils ($\bar{x}$ ± *SE*, *n* = 264) subjected to different grazing intensities. Different letters (A, B, and C) indicate significant differences (*p* < .05)

There were gender effects in the competing models in all three sites (Table S2), so we analyzed the effect of grazing on survival by gender. Almost all competing models included the effects of grazing on both males and females (Table 2). In all of the grazing treatments, male survival probability had a time effect, and survival probability in late autumn was lower than at other times. Males had a greater survival probability in the overgrazing and lightly grazed sites than in the grazing exclusion sites (Figure 4), but the opposite was found for females (Figure 5). Because the survival model is built in a long‐term continuous marking process, the model may include a different age stage of a midday gerbil, so the model cannot analyze the survival status at a certain point in time. At the same time, there is no significant difference in age structure between them.

**TABLE 2:** Models of local survival probabilities (φ) in male and female midday gerbils using Cormack–Jolly–Seber (CJS) models respective

Genders	No.	Model^a^	QAICc^b^	ΔQAICc^c^	QAICc Weights^d^	M‐Likelihood^e^	np^f^	QDev.^g^
Male	1	***φ*(g)*p*(g)**	**848.586**	**0.000**	**0.480**	**1.000**	**8**	**393.709**
	2	***φ*(t)*p*(g)**	**849.707**	**1.120**	**0.274**	**0.571**	**45**	**313.652**
	3	*φ*(.)*p*(g)	850.791	2.205	0.159	0.332	5	402.056
	4	*φ*(t)*p*(.)	852.983	4.397	0.053	0.111	42	323.909
	5	*φ*(g + t)*p*(g)	854.167	5.580	0.029	0.061	48	311.055
	6	*φ*(g*t)*p*(g*t)	2,021.429	1,172.843	0.000	0.000	328	141.979
Female	1	***φ*(g)*p*(g)**	**799.917**	**0.000**	**0.987**	**1.000**	**8**	**381.019**
	2	*φ*(g)*p*(.)	808.587	8.670	0.013	0.013	5	395.831
	3	*φ*(g*t)*p*(g*t)	2,002.300	1,202.383	0.000	0.000	328	158.829

Estimation of the recapture parameters (*p*) has an additional time effect or interaction among time, sex, and grazing. No. indicates model rank;

QAICc, quasi‐likehood corrected Akaike information criterion (AIC) for small sample size and overdispersion (1.04);

ΔQAICc, difference between model QAICc and minimum QAICc;

QAICc weight, relative strength of evidence for a model within the set of models computed; np, number of parameters;

Model likelihood, relative strength of evidence for a model within the set of models computed;

number of parameters;

Quasi deviance. Only models with ΔQAICc < 7 are shown. Models were ranked by QAICc, and the most parsimonious models are in boldface type. T, time interval; S, sex; G. grazing. The interactions between parameters and additive effects are noted with asterisks (×) and addition signs (+), respectively.

### 3.4 Sex ratio (females/males)

**FIGURE 6:**
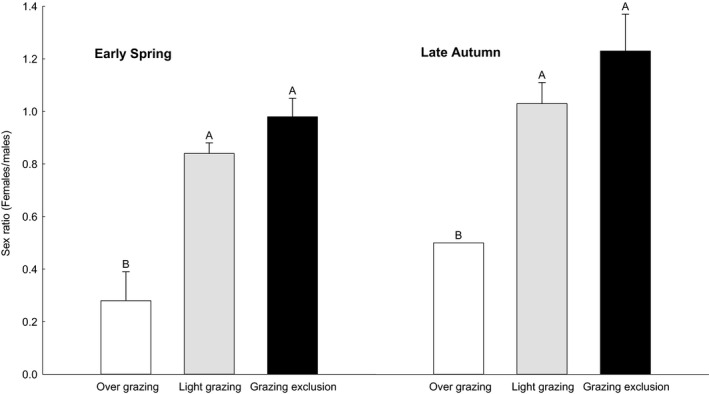
Sex ratio (females/males, $\bar{x}$ ± *SE*) of midday gerbils captured from overgrazing, light grazing, and grazing exclusion sites in early spring and late autumn, 2006 to 2010 in Alxa Left Banner, Inner Mongolia, China. Different letters (A, B, and C) indicate significant differences (*p* < .05) between overgrazing, light grazing, and grazing exclusion sites, according to Tukey's test.

The sex ratio of midday gerbils captured showed a significant difference between the light grazing or grazing exclusion areas and the overgrazing areas in the early spring (*F*_2,6_ = 41, *p* < .05) and late autumn (*F*_2,6_ = 9, *p* < .05). There was no significant difference between the light grazing and grazing exclusion areas (Figure 6).

### 3.5 Food resources

**FIGURE 7:**
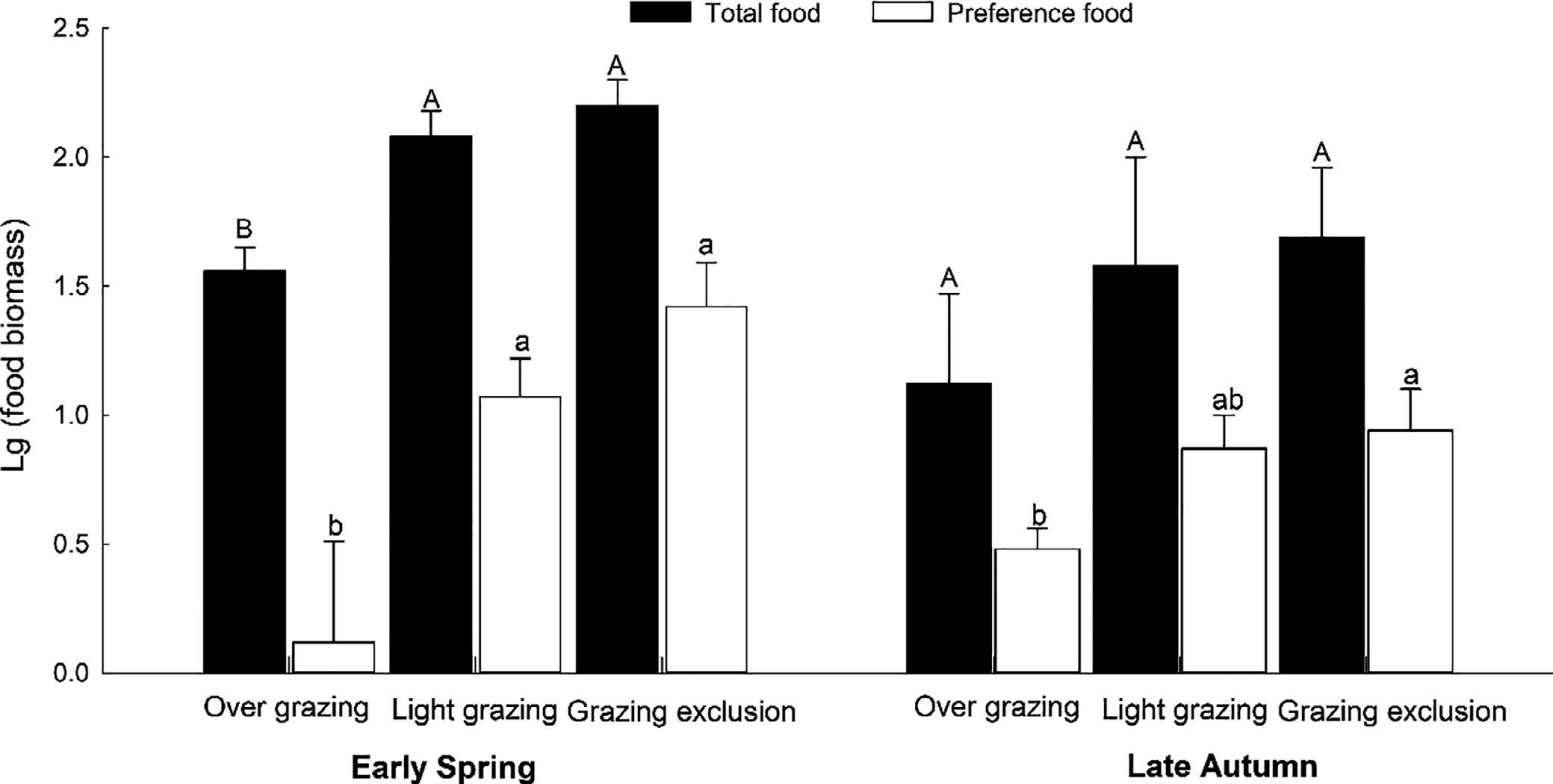
Total food biomass (TFB, $\bar{x}$ ± *SE*) and preferred food biomass (PFB, $\bar{x}$ ± *SE*) in overgrazing (OG), light grazing (LG), and grazing exclusion (EG) sites in April (early spring(a)) and October (late autumn (b)). Different letters (A, B, C or a, b, c) indicate significant differences (*p* < .05)

**FIGURE 8:**
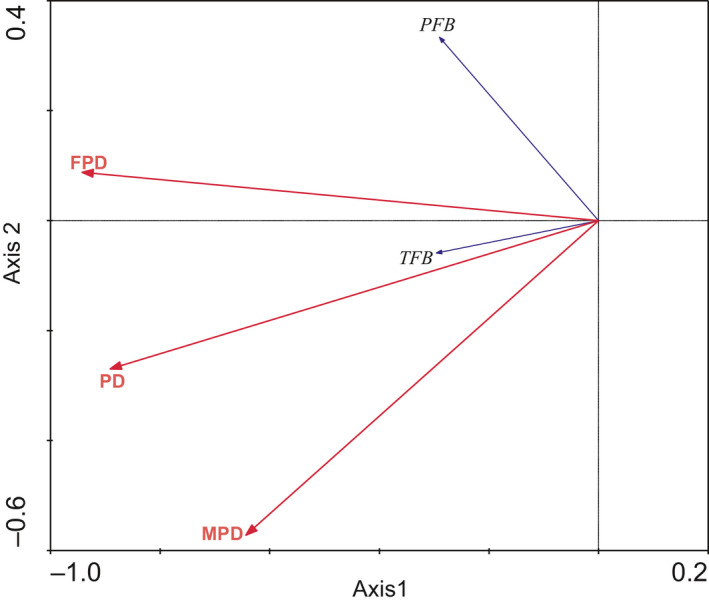
Result of RDA analysis: the diagram depicts the relation between gerbil population density (PD), female gerbils population density (FPD), male gerbil population density (MPD) and plant variables. Only variables with significant impacts are depicted (*p* < .05). Food biomass (TFB, PFB) and midday gerbil abundance refers to data in the experiment areas from 2008 to 2010

In early spring, grazing exclusion and light grazing areas significantly enhanced TFB (*F*_2,19.57_ = 3.85, *p* < .05) and PFB (*F*_2,19.55_ = 4.2 *p* < .05) compared to the overgrazing areas (Figure 7). The year had a significant effect on TFB in early spring (*F*_4,22_ = 5.533, *p* = .003), but there is no interyear difference in PFB (*F*_4,22_ = 0.643, *p* = .683) in early spring. In late autumn, there was no significant difference in TFB among all treatment areas (*F*_2,18.44_ = 1.12, *p* = .35), but PFB in the grazing exclusion areas was significantly greater than in the overgrazing areas (*F*_2,27_ = 6.07, *p* < .01, Figure 7). There is no interyear difference on the TFB (*F*_4,22_ = 1.66, *p* = .194) and PFB (*F*_4,22_ = 1.219, *p* = .331) in late autumn. There was a significant impact of TFB and PFB on female density and gerbil population density. TFB affected male density, but PFB has no effect on male population density (Figure 8).

## 4 DISCUSSION

Winter is a bottleneck period for mammalian nonhibernators due to food shortage and harsh environmental conditions at high northern latitudes (Coltrane & Barboza, 2010; Solonen, 2006). The overwinter survival of gerbils is affected by many mortality risks. The mortality risk faced by wild animal populations depends on their intrinsic conditions (e.g., age‐related experience, body condition, immune system status, and population structure), but the intrinsic conditions are always influenced by many external conditions (e.g., meteorological conditions, predation risk, food availability, and disturbance) (Chantepie et al., 2015; Forslund & Pärt, 1995; Théoret‐Gosselin et al., 2015). In our study, all treatments were applied under the same meteorological conditions, so meteorological conditions do not play an important role in our study. A previous study has shown that the midday gerbil is a typical arid‐area rodent, which prefers habitats with high vegetation coverage in extremely arid desert area (Fu et al., 2004), which was also confirmed by our finding of greater density in grazing exclusion and light grazing areas than in overgrazing areas. Other studies had shown that low grazing can have negative effect on rodents in range lands. However, the vegetation and climate of the abovementioned study area are different from the situation in this study area (Tabeni & Ojeda, 2005; Tchabovsky et al., 2019). The environment in above study area will change greatly, and grassland types will have succession phenomena when long‐term reduced grazing intensity, and different species show different coping strategies (Jones et al., 2003). Our result is consistent with former studies suggesting that grazing exclusion may increase the number of rodents (Heske & Campbell, 1991; Keesing, 1998b; Rosi et al., 2009). Our models show a significant effect of grazing on overwinter survival. The body mass, growth rate, and food availability of gerbils differed between the overgrazing and grazing exclusion areas. Therefore, there are three main clusters of factors influencing the population characteristics of gerbils.

The first is food availability. The results of this study indicate that food availability is highest in the grazing exclusion areas, and significantly higher than in light grazing and overgrazing areas. Adequate food availability results in faster weight accumulation and larger body weight of gerbils in grazing exclusion areas. Our result is consistent with previous studies which observed heavier individuals in grazing exclusion plots than in grazing plots (Bueno et al., 2012; Keesing, 1998b). Previous studies suggested that food addition in the growing or breeding season can increase population densities of rodents through the enhancement of recruitment and reproduction, but does not improve survival, mainly due to the trade‐off between reproduction and winter survival in harsh environments (Andrzejewski, 1975; Doonan & Slade, 1995; Taitt & Krebs, 1981). Winter food availability would limit winter survival of voles (Johnsen et al., 2017) and densities of breeding mouse the following spring. Thus, increased food availability during winter would result in higher breeding bird densities the following spring. A study on Mongolian gerbils (*Meriones unguiculatus*) found that group sizes were larger in food supplemented chambers than in unsupplemented chambers (Liu et al., 2011). This is consistent with our results. Grazing exclusion areas, similar to food addition, held higher TFB and PFB than in the overgrazing areas across the whole year. Therefore, compared to the light grazing and overgrazing plots, body mass, body mass growth rates, and the density of gerbils were higher in the grazing exclusion sites. Other study have predicted that in long‐term perspective, intense grazing as well as low grazing can cause qualitative changes in vegetation and habitat structure, which may have long‐term positive or negative effects on rodents depending on their ecological requirements. In particular, negative long‐term effects of low grazing on population abundance and demographic parameters (including survival) were shown for gerbils (Tchabovsky et al., 2016, 2019). In above cases, the gerbils demographic parameters keep a relatively negative relationship with the light grazing. The main reason is that the type of grassland has changed from desert to steppe and the gerbils cannot adapt to this living environment.

The second cluster of factors affects reproduction. Previous research predicted that sufficient fat and body mass would cause a higher overwinter survival both in male and female hibernating rodents (Schorr et al., 2009). However, female *Microtus oeconomus* showed a positive correlation between probability of survival and body mass, while no such effect was observed in males (Korslund, 2006), and females showed lower survival rates after breeding (Aars & Ims, 2002). A previous study indicated that both male and female meridian gerbils can form stable mate preferences, with behavioral characteristics of monogamous species (Zhang et al., 2016). The female gerbil had enough energy for mating and reproduction due to their higher body mass and higher PFB in grazing exclusion areas. Therefore, the female gerbils had higher overwinter survival in grazing exclusion sites. This study indicated a lower sex ratio (females/males) in overgrazing areas than in grazing exclusion areas. Male gerbils thus allocated less energy and time for mating behavior, and they need less food to support their daily activities due to higher survival in the overgrazing sites.

The third aspect is predation risk. In our study, male gerbils had higher overwinter survival in overgrazing areas than in lightly grazing and grazing exclusion areas, but female gerbils did not, showing higher survival properties in grazing exclusion areas. The removal of livestock could increase plant cover, thereby reducing the exposure of small mammals to their avian predators (Peles & Barrett, 1996). Rodents may be at risk of avian predation in exposed microhabitats, but were more susceptible to predation by snakes in sheltered microhabitats (Bouskila, 1995; Kotler et al., 1991, 1993). Gerbils are nocturnal rodents, so they are more likely to be hunted by night‐time predators such as foxes, snakes, and weasels, rather than birds. In overgrazing plots, male gerbils spend less time out of the nest due to their lower reproductive investment, thereby facing lower predation risk. Therefore, they would face higher predation risk in the grazing exclusion sites. Thus, gerbils showed higher survival ability in overgrazing than in grazing exclusion sites.

In our study, a lower population density occurs in late autumn and a higher in spring in our experiment. There are two main reasons for this result. The first reason is that the autumn midday gerbils are in a period of concentrated storage of food, so they face a higher predation risk, and the population will decline. Second, because autumn is a peak period for midday gerbils’ reproduction, their pups grow into adults in the spring of the second year, increasing the population of midday gerbils, so the number of midday gerbils in spring is generally higher than the previous year's late autumn. This result also shows that winter has a great influence on the population of small rodents.

We concluded that grazing influenced midday gerbil population density and body mass negatively due to reduced food availability in late autumn and early spring. For female gerbils, overwinter survival was higher in grazing exclusion sites than in light grazing sites and overgrazing sites, while male gerbils showed higher survival in overgrazing than in lightly grazed sites and grazing exclusion sites. Further research is required on the factors affecting survival rates, with a focus on other factors that differentially affect male and female survival rates. These factors may include adjustments within their populations or other abiotic factors, such as climate.

## CONFLICT OF INTEREST

The authors declare that they have no conflict of interest.

## AUTHOR CONTRIBUTION

**Su‐Wen Yang:** Data curation (lead); Methodology (lead); Writing‐original draft (lead). **Shuai Yuan:** Funding acquisition (lead); Writing‐review & editing (lead). **Xiao‐Dong Wu:** Project administration (equal); Writing‐review & editing (equal). **Rong Zhang:** Methodology (supporting); Writing‐original draft (supporting). **Xiu‐Xian Yue:** Investigation (supporting); Writing‐review & editing (supporting). **Yu Ji:** Methodology (supporting); Software (supporting). **Lin‐Lin Li:** Data curation (supporting); Software (supporting). **Xin Li:** Methodology (supporting); Software (supporting). **He‐Ping Fu:** Investigation (equal); Methodology (equal); Writing‐review & editing (equal).

## Supporting information

Supplementary Material

## Data Availability

Primary data including total data have been deposited in the Dryad Repository. https://doi.org/10.5061/dryad.dncjsxkvv.
