# Supplementary material for: The effect of grazing on winter survival of midday gerbil (Meriones meridianus) of different genders
Source: Ecol Evol. 2020 Oct 15;10(21):12395–406. doi: 10.1002/ece3.6870 (PMC7845001; doi:10.1002/ece3.6870)
Supplement: Supplementary file 1 — Supplementary Material [file ECE3-10-12395-s001.doc]

**SUPPLEMENTARY MATERIALS**

# Feeding experiment of midday gerbils

Gerbils was live-trapped in each season (autumn and spring) from a desert habitat at a distance from the study sites and fasted for 8 h before placing them into the cages at dusk. A total of 12 gerbils were used (six in the autumn of 2017, six in spring of 2017), weighing on average 50.75±4.77 g (Mean±SD). Each gerbil was randomly assigned to one cage. We provided 20 g of each plant species to each subject in 100-mm petri dishes placed in a randomized array with one species per dish. We fed gerbils every 3 hours, about 2-3 times per night. Ten to twelve plant species were fed at each time. The plant species fed for each gerbil was the same each night, but plant species were arranged randomly for every gerbil at same time. We collected the remaining plants (including those cached throughout the cages) and plant remnants, separated and weighed them by species, when we put the new plant species in. Same plant species with 20g weight were placed outside the cages as a control group to determine water loss. We calculated species composition of consumed plants by subtraction.

Weight of a consumed plant species=20-remaining weight-water loss

Gerbils spent an entire night in the cages with access to the plant food and were released the next morning at the point of capture. Nine trials (5 males and 4 females) were conducted in the autumn of 2015 and 6 (3 males and 3 females) in spring of 2016. Then, the preference index (PI) was calculated ([Batzli and Pitelka 1983](../../../../D:%5Csam%20folder%5C%E8%A2%81%E5%B8%85%5C%E7%A0%94%E7%A9%B6%E7%94%9F%E8%AE%BA%E6%96%87%E6%9D%90%E6%96%99%5C%E6%9C%80%E8%BF%91%E8%A6%81%E5%AE%8C%E6%88%90%5CJOZ%5C2015%E5%B9%B42%E6%9C%88%E4%BF%AE%E6%94%B9%5C%E9%99%84%E8%A1%A82.docx#_ENREF_4)). (PI=proportion in diet/proportion of available forage). PI will be greater than one if the preference is positive and less than one if the preference is negative. Preferred food and potential food resources were divided by the preference index (PI) .

Table S1 Preference index of midday gerbil

Note: Bolds represent preference food.

| Plant Species | Preference index | |
| --- | --- | --- |
|  | April | September |
| *Sarcozygium xanthoxylon* | **2.15** | 0.95 |
| *Pennisetum centrasiaticum* | - | **1.88** |
| *Nitraria tangutorum* | - | 0.79 |
| *Artemisia sphaerocephala* | - | 0.07 |
| *Corispermum mongolicum* | **3.46** | 0.90 |
| *Salsola collina* | - | **1.95** |
| *Cynanchum thesioides* | **1.80** | **1.16** |
| *Caragana brachypoda* | 0.72 | 0.87 |
| *Cynanchum chinense* | 0.39 | **1.23** |
| *Setaria viridis* | - | **2.06** |
| *Scorzonera* divaricata | - | 0.72 |
| *Reaumuria songarica* | - | 0.68 |
| *Hedysarum* scoparium | - | 0.24 |
| *Achnatherum splendens* | **2.49** | 0.46 |
| *Micropeplis arachnoidea* | - | **2.74** |
| *Sonchus* arvensis | - | **1.56** |
| *Echinops* gmelini | - | **1.14** |
| *Phragmites australis* | **1.14** | 0.58 |
| *Peganum harmala* | - | 0.66 |
| *Oxytropis aciphylla* | 0.09 | **1.04** |
| *Artemisia xerophytica* | 0.21 | 0.30 |
| *Caragana korshinskii* | - | 0.02 |
| *Cynanchum hancockianum* | **1.39** | 0.39 |
| *Panzeria lanata var*.alaschanica | 0.59 |  |
| *Astragalus* galactites | **1.19** | 0.64 |
| *Allium mongolicum* | **1.58** | 0.28 |
| *Ammopiptanthus mongolicus* | - | 0.92 |
| *Agriophyllum pungens* | **1.27** | **1.86** |
| *Atraphaxis frutescens* | **1.70** | **1.42** |
| *Ixeris denticulata* | **1.58** | - |
| *Haloxylon ammodendron* | 0.10 | 0.95 |
| *Carex Stenophylloides* | **1.49** | **2.09** |
| *Asparagus cochinchinensis* | 0.52 | **1.06** |
| *Plantago lessingii* | **1.21** | **2.74** |
| *Ceratoides intramongolica* | **3.27** | **1.07** |
| *Bassia dasyphylla* | - | **1.54** |
| *Convolvulus ammannii* | 0.93 | **1.41** |
| *Cleistogenes songorica* | - | **3.90** |
| *Stipa glareosa* | - | 0.92 |

Potential food resource samples were visually found and manually collected randomly and concomitantly to gerbil near or inside the trapping sites. Leaves, stems, flowers, fruits, and seeds of 22 plant species in spring and 37 plant species in autumn were collected. To determine a preference food for these plant species, we conducted cafeteria style preference trials in 6 to 9 wire-mesh cages (100 cm × 50 cm × 30 cm) with attached wooden nest boxes in the autumn and spring of 2017. The bottom of each cage consisted of a fine screen underlain by a wire-mesh floor constructed so that sand could be drained out, leaving plants and plant remnants on the screen for collection. The cages were placed in a laboratory not far away from our study sites.

# About the CJS models

Over-dispersion of the general model [ϕ(Sex×Time) p(Sex×Time)] for sex effects was tested using the bootstrap goodness-of-fit method within MARK with 500 iterations. The test showed evidence of over-dispersion with the variance inflation factor c-hat of 1.20 (*P*=0.13), 1.37 (*P*=0.06) and 1.95 (*P*=0.12, Table S1). Thus, we used quasi Akaike information criterion corrected for small sample size (QAICc) to select the most parsimonious and competing models of gerbil monthly survival . The most parsimonious model is the model with the lowest QAICc among all candidate models considered. A model with ∆QAICc<2 was considered as a competing model . We built 15 candidate models including all possible models for sex effects in each treatment respectively, sex and their interaction on survival probabilities and all possible models of the effects of sex, time, and sex-time interaction on recapture probabilities. If a model including sex effects on survival probabilities was the most parsimonious model or a competing model, we concluded that survival of gerbils was significantly different between genders.

Table S2 Goodness-of-fit test of general models for local survival probabilities (φ) in midday gerbils with different genders

| Treatment | Model | Observed deviance | mean of the simulated deviances | $\mathop{\text{c}}\limits^{\wedge}$ | *P* |
| --- | --- | --- | --- | --- | --- |
| Grazing exclusion | φ(s*t)p(s*t) | 161.24 | 134.03 | 1.20 | 0.13 |
| Light Grazing | φ(s*t)p(t) | 68.93 | 50.45 | 1.37 | 0.06 |
| Over Grazing | φ(s*t)p(s*t) | 8.86 | 4.55 | 1.95 | 0.12 |

Note: Estimation of the recapture parameters (P) has an interaction among time and sex. T, time interval; S, sex. The interactions between parameters were noted with an asterisk (*).$\mathop{\text{c}}\limits^{\wedge}$:variance inflation factor; *P*: The possibility of simulated deviance larger than observed deviance.

Table S3 Gender effects on local survival probabilities (φ) of midday gerbils subjected to different grazing intensity using CJS models

| Grazing intensity | Model | QAICc | ΔQAICc | QAICc Weights | M-Likelihood | np | QDev. |
| --- | --- | --- | --- | --- | --- | --- | --- |
| Grazing  exclusion | ***φ*(s)*p*(.)** | **545.946** | **0.000** | **0.541** | **1.000** | **3** | **255.903** |
|  | ***φ*(s)*p*(s)** | **546.585** | **0.639** | **0.393** | **0.727** | **4** | **254.504** |
|  | *φ*(.)***p***(.) | 550.883 | 4.937 | 0.046 | 0.085 | 2 | 262.869 |
|  | *φ*(.)***p***(s) | 552.588 | 6.642 | 0.020 | 0.036 | 3 | 262.545 |
|  | *φ*(s*t)*p*(s*t) | 709.481 | 163.535 | 0.000 | 0.000 | 108 | 134.034 |
| Light grazing | ***φ*(.)***p***(.)** | **261.768** | **0.000** | **0.526** | **1.000** | **2** | **132.989** |
|  | ***φ*(s)***p***(.)** | **263.725** | **1.957** | **0.198** | **0.376** | **3** | **132.900** |
|  | φ(.)*p*(s) | 263.757 | 1.989 | 0.195 | 0.370 | 3 | 132.932 |
|  | φ(s)*p*(s) | 265.527 | 3.759 | 0.080 | 0.153 | 4 | 132.642 |
|  | φ(s*t)*p*(t) | 366.274 | 104.506 | 0.000 | 0.000 | 71 | 47.903 |
| Over grazing | ***φ*(.)*p*(.)** | **41.000** | **0.000** | **0.489** | **1.000** | **2** | **24.920** |
|  | ***φ*(s)*p*(.)** | **42.708** | **1.707** | **0.208** | **0.426** | **3** | **24.453** |
|  | *φ*(.)*p*(s) | 43.174 | 2.174 | 0.165 | 0.337 | 3 | 24.920 |
|  | *φ*(s)*p*(s) | 43.525 | 2.525 | 0.138 | 0.283 | 4 | 23.034 |
|  | *φ*(s*t)*p*(t) | 138.553 | 97.552 | 0.000 | 0.000 | 33 | 4.541 |

Note: Estimation of the recapture parameters (P) has an interaction among time and sex. No., Model rank; QAIC_c_, quasi-likehood corrected Akaike information criterion (AIC) for small sample size and over-dispersion; ΔQAIC_c_, difference between model QAIC_c_ and minimum QAIC_c_; QAIC_c_ weight, relative strength of evidence for a model within the set of models computed; np, number of parameters; Model likelihood, relative strength of evidence for a model within the set of models computed. Only models with ΔQAICc<7 were shown. Models were ranked by QAIC_c_, and the most parsimonious models are in boldface type. T, time interval; S, sex; G. grazing. The interactions between parameters are noted with an asterisk (*).
